# The effect of Er:YAG laser irradiation on the bond stability of self-etch adhesives at different dentin depths

**DOI:** 10.1007/s10103-017-2194-x

**Published:** 2017-03-30

**Authors:** Muhammet Karadas, İpek Çağlar

**Affiliations:** 1 Department of Restorative Dentistry, Recep Tayyip Erdogan University, Rize, Turkey; 2 Department of Prosthetic Dentistry, Recep Tayyip Erdogan University, Rize, Turkey

**Keywords:** Er:YAG laser, Bond stability, Self-etch adhesive, Dentin, Micro-shear bond strength

## Abstract

The aim of this study was to evaluate the effect of Er:YAG laser irradiation on the micro-shear bond strength of self-etch adhesives to the superficial dentin and the deep dentin before and after thermocycling. Superficial dentin and deep dentin surfaces were prepared by flattening of the occlusal surfaces of extracted human third molars. The deep or superficial dentin specimens were randomized into three groups according to the following surface treatments: group I (control group), group II (Er:YAG laser; 1.2 W), and group III (Er:YAG laser; 0.5 W). Clearfil SE Bond or Clearfil S^3^ Bond was applied to each group’s dentin surfaces. After construction of the composite blocks on the dentin surface, the micro-shear bond testing of each adhesive was performed at 24 h or after 15,000 thermal cycles. The data were analyzed using a univariate analysis of variance and Tukey’s test (*p* < 0.05). Laser irradiation in superficial dentin did not significantly affect bond strength after thermocycling (*p* > 0.05). However, deep-dentin specimens irradiated with laser showed significantly higher bond strengths than did control specimens after thermocycling (*p* < 0.05). Thermocycling led to significant deterioration in the bond strengths of all deep-dentin groups. The stable bond strength after thermocycling was measured for all of the superficial-dentin groups. No significant difference was found between the 0.5 and 1.2 W output power settings. In conclusion, the effect of laser irradiation on the bond strength of self-etch adhesives may be altered by the dentin depth. Regardless of the applied surface treatment, deep dentin showed significant bond degradation.

## Introduction

The basic mechanism of adhesion between tooth substrate and adhesive bonding agents is based on an exchange process. Minerals from the hard tissues of teeth are replaced by adhesive monomers that effectively create a micromechanical interlock after polymerization [1]. Before the bonding agent is applied, the standard procedure for better bonding is the etching of enamel with phosphoric acid [2]. However, the moist and organic nature of dentin has prevented the development of a reliable, durable resin-dentin bonding. Bonding stability to dentin depends on the formation of a homogenous and compact hybrid layer. The degradation of the dentin-bonding interface is caused by hydrolysis and proteolytic breakdown of the collagen fibril components of the hybrid layer or by hydrolytic degradation of the hybrid layer’s adhesive components [3, 4].

Self-etch adhesives demineralize the dentin during the process of resin infiltration, potentially ensuring a more complete resin infiltration [5]. Several studies have determined nanoleakage under the hybrid layer of self-etch adhesive bonding agents, casting doubt on whether complete resin infiltration occurs [6, 7]. Van Landuyt et al. stated that self-etch adhesive bonding agents decreased the bond strength to dentin after immersion in water for 6 months. Also, they observed that self-etch adhesives failed often under hybrid layer at dentin. These failures have been linked to insufficient encapsulation of the surface smear layer [8].

The introduction of laser technology in dentistry offers a new alternative to the standard method for cavity preparation, allowing removal of caries with both high- and low-speed rotary instruments. Among the different laser types used in dentistry, Er:YAG and Er,Cr:YSGG laser devices have performed best with use in dental hard tissues, as their wavelengths are close to the peak with respect to the hydroxyl groups and water absorption [9, 10]. Er:YAG laser reacts on dental hard structures without leading to more severe thermal side effects or injury to the pulp (such as melting or cracking) when it is continuously used with proper amount of water cooling and correct treatment parameters [11]. Dentin surfaces irradiated with an Er:YAG laser have shown microstructural alterations with a rough appearance and opened dentinal tubules, without a smear layer and demineralization, suggesting that this process provides a better substrate for adhesive restorations [12]. Most studies have examined the bond strengths of adhesive materials to dentin treated with Er:YAG laser, comparing them to their bond strengths to free-hand-irradiated dentin. However, the bond strengths reported with dentin treated with Er:YAG laser have not been consistent.

The energy density, pulse energy, and frequency of Er:YAG laser settings advised for cavity preparation are the most important parameters to ablate dental tissues [13]. The use of erbium lasers for tooth-surface conditioning requires the use of lower energy densities than those used for cavity preparation [14]. However, the precise laser parameters have not yet been determined for optimized resin–dentin bonding strength during tooth-surface conditioning. It is believed that several factors, including the output power of the laser, the adhesive system applied, and the acid etching affect the quality of bonding to laser-irradiated dentin [15–17].

Many researchers have evaluated the immediate bond strengths of adhesive materials to dentin irradiated using the Er:YAG laser. However, some studies have emphasized that immediate bonding strength is not always associated with long-term bonding results [18]. The limited studies that exist on the bonding durability of dentin treated with Er:YAG laser have controversial results [19, 20]. No information exists regarding the bonding stability of self-etch adhesive materials at various depths of laser-irradiated dentin. Therefore, the aim of the present study was to assess the effect of Er:YAG laser irradiation on bond strengths of one-step and two-step self-etch adhesives to irradiated superficial dentin and deep dentin after thermocycling. The null hypothesis to be tested was that the surface condition of teeth after use of an Er:YAG laser at different dentin depths did not affect the bond strength of self-etch adhesive bonding agents after thermocycling.

## Materials and methods

### Specimen preparation and surface treatments

**Fig. 1:**
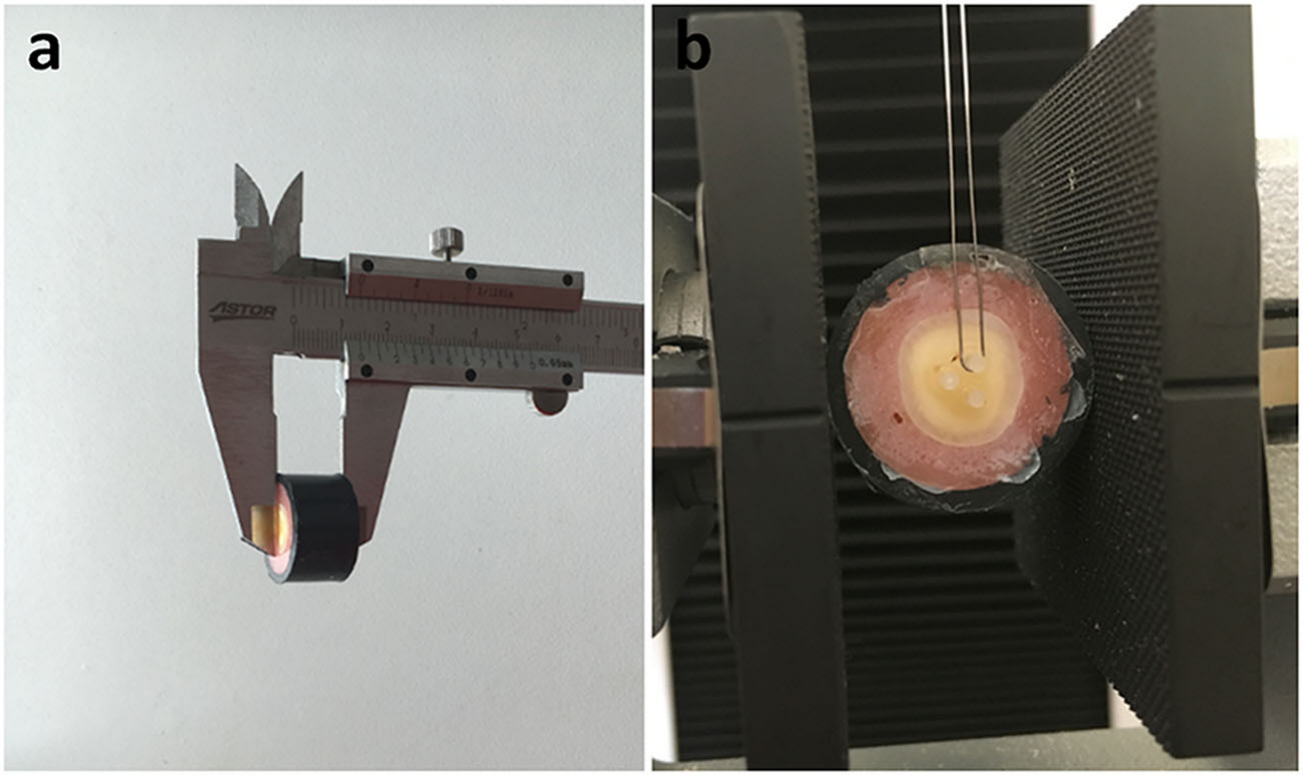
Specimen preparation and experimental design used to measure micro-shear bond strength

One hundred and twenty sound human third molar teeth were used in this study. The teeth were immersed for 2 weeks in chloramine T solution (0.5%) at room temperature. The roots were sectioned from the crowns at the cemento-enamel junction with a diamond bur under a water spray. Each crown was mounted in cold-cure acrylic with the occlusal surface parallel to the base. The occlusal surface was flattened with 240-grit sandpaper under a stream of water on a polishing machine (LaboPol-25, Struers, Denmark) until all of the enamel was completely removed. The absence of enamel on the occlusal surface was confirmed with a stereomicroscope (SZ 40; Olympus, Tokyo, Japan) and the dentin level obtained termed “superficial dentin.” To create the deep-dentin surfaces, half of the specimens were additionally abraded until a depth of 1.5 mm from the superficial dentin was reached; depth was measured using a metal caliper (Fig. 1a) [5]. Finally, dentin surfaces were prepared with 600-grit sandpaper for 30 s to form standardized smear layers.

The superficial dentin or deep-dentin specimens were randomly divided into three groups according to the following surface treatments:

- Group I: The control group was exposed to polishing only with abrasive papers.
- Group II: The Er:YAG laser (LightWalker; Fotona, Ljubljana, Slovenia) was used to condition the dentin surfaces, with a pulse frequency of 10 Hz and a power output of 1.2 W under water cooling set for 5 ml/min. The LightWalker laser has a wavelength of 2940 nm and a pulse duration of 150 μs. Energy density was 18.9 J/cm^2^. The HCO_2_-N hand-piece was kept perpendicular to the dentin surface, at a distance of 10 mm. The spot size of the laser beam was 0.9 mm. To standardize the distance between the dentin surface and the laser tip, a marked bur was fixed to the head of the hand-piece using a prepared acrylic device. Manual irradiation was performed by scanning all of the dentin surfaces in both the vertical and the horizontal direction.
- Group III: Dentin surface conditioning was performed as defined above in Group II, but with a pulse frequency of 5 Hz and a power output of 0.5 W. Energy density was 15.7 J/cm^2^.

### Bonding procedures

All prepared surfaces in each group were bonded with Clearfil SE Bond (two-step self-etch adhesive: apply primer for 20 s, dry with mild air flow; apply bond and air flow for 5 s) or Clearfil S^3^ Bond (one-step self-etch adhesive: apply bond for 20 s, dry with high-pressure air flow for least 5 s) according to the manufacturer instructions (Kuraray Inc., Osaka, Japan). Three Tygon tubes (Saint-Gobain Corporation; Courbevoie, France) of 1-mm internal diameter were placed over the central area of the dentin surface. Each adhesive agent was light-cured for 10 s using a light-emitting diode unit (VALO Cordless; Ultradent, South Jordan, Utah, USA) with a power output of 1000 mW/cm^2^, checked by a radiometer (TREE, model TR-P004, China). Bulk-fill flowable resin composite (3 M ESPE, St. Paul, Minnesota, USA) was filled into Tygon tubes up to a height of 1.5 mm and light-polymerized for 40 s with the same curing unit. The specimens with bonded composites were stored for 24 h in distilled water at 37 °C prior to the removal of the plastic Tygon tubes. For each adhesive agent, half of the specimens were subjected to 15,000 thermal cycles that were split between water baths (dwell time, 15 s) at 5 and 55 °C. The remaining specimens were immediately subjected to bond testing. This study covered 24 subgroups by combining 2 testing conditions (24 h and thermocycling) × 2 adhesive agent subgroups × 3 surface treatment groups × 2 different dentin surfaces.

### Micro-shear bond strength testing

Each bonded specimen was mounted on a universal testing machine (Model 3344, Instron Corporation, Norwood, MA, USA). An orthodontic-loop wire was placed around the resin composite cylinder (Fig. 1b), followed by shear forces being applied to the adhered resin composite cylinders at a crosshead speed of 1 mm/min until failure. Shear bond strength in megapascals (MPa) was calculated from the maximum load of failure in Newton divided by the bonding surface area (пr^2^ = 0.785 mm^2^). Following testing, the fractured surfaces were examined under a stereomicroscope at ×40 magnification. The failure mode of each specimen was recorded in one of three following types: adhesive (failure at the adhesive interface), cohesive (failure in dentin or resin composite), and mixed (combined cohesive and adhesive failure).

### Statistical analysis

Descriptive statistics for groups were calculated using the SPSS 18.0 software package (Chicago, IL, USA). The data distribution and equality of the group variances were analyzed using Levene’s test and the Kolmogorov–Smirnov test. A univariate analysis of variance and Tukey’s post-hoc test were used to compare the data. Failure mode data were statistically analyzed with the chi-square test, and *p* < 0.05 was considered statistically significant.

## Results

The micro-shear bond strength results at 24 h and after thermocycling are shown in Tables 1 and 2, respectively. At the 24-h testing time, the micro-shear bond strength of Clearfil SE Bond to both superficial dentin and deep dentin was significantly higher than that of Clearfil S^3^ Bond in Group I (control group). No significant difference was found between the bond strengths of adhesive agents in each laser group (Group II and III), regardless of testing time and dentin type. For Clearfil S^3^ Bond at 24 h, the bonding strength to superficial dentin and deep dentin increased when laser irradiation was applied. The laser irradiation did not statistically affect the bond strength of Clearfil SE Bond to the superficial dentin when compared to the control group at 24 h, but the bond strength to deep dentin improved.

**Table 1:** Mean micro-shear bond strengths (standard deviations) and failure modes of experimental groups at 24 h

Group	Adhesive	Number	Superficial dentin	Deep dentin		
			Mean (SD)	Failure modes	Mean (SD)	Failure modes
Group 1	Clearfil SE Bond	15	33.48 (8.1) abA	9/2/4	17.42 (4.1) bB	12/2/1
	Clearfil S^3^ Bond	15	23.11 (5.7) cA	11/0/4	9.90 (2.9) cB	15/0/0
Group 2	Clearfil SE Bond	15	40.93 (7.1) aA	7/2/6	28.51 (4.3) aB	10/1/4
	Clearfil S^3^ Bond	15	33.57 (5.9) abA	9/3/3	26.20 (5.8) aB	10/0/5
Group 3	Clearfil SE Bond	15	29.49 (13.4) bcA	10/3/2	31.33 (6.7) aA	11/2/2
	Clearfil S^3^ Bond	15	30.02 (6.1) bcA	8/2/5	28.46 (4.9) aA	10/3/2

Different lowercase letters indicate significant difference in the vertical column (*p* < 0.05). Different uppercase letters indicate significant difference in the horizontal row (*p* < 0.05). Failure mode (adhesive/cohesive/mixed)

**Table 2:** Mean micro-**s**hear bond strengths (standard deviations) and failure modes of experimental groups after thermocycling

Group	Adhesive	Number	Superficial dentin	Deep dentin		
			Mean (SD)	Failure modes	Mean (SD)	Failure modes
Group 1	Clearfil SE Bond	15	37.13 (8.1) aA	9/2/4	8.68 (4.1) bB	14/1/0
	Clearfil S^3^ Bond	15	29.80 (9.3) aA	9/1/5	3.31 (2.5) bB	15/0/0
Group 2	Clearfil SE Bond	15	38.61 (8.1) aA	6/2/7	22.53 (3.7) aB	9/2/4
	Clearfil S^3^ Bond	15	36.02 (11.5) aA	8/2/5	16.93 (3.9) aB	11/0/4
Group 3	Clearfil SE Bond	15	29.24 (10.6) aA	8/2/5	23.83 (7.5) aB	12/0/3
	Clearfil S^3^ Bond	15	31.83 (8.2) aA	7/2/6	19.54 (4.3) aB	13/0/2

Different lowercase letters indicate significant difference in the vertical column (*p* < 0.05). Different uppercase letters indicate significant difference in the horizontal row (*p* < 0.05). Failure mode (adhesive/cohesive/mixed)

**Fig. 2:**
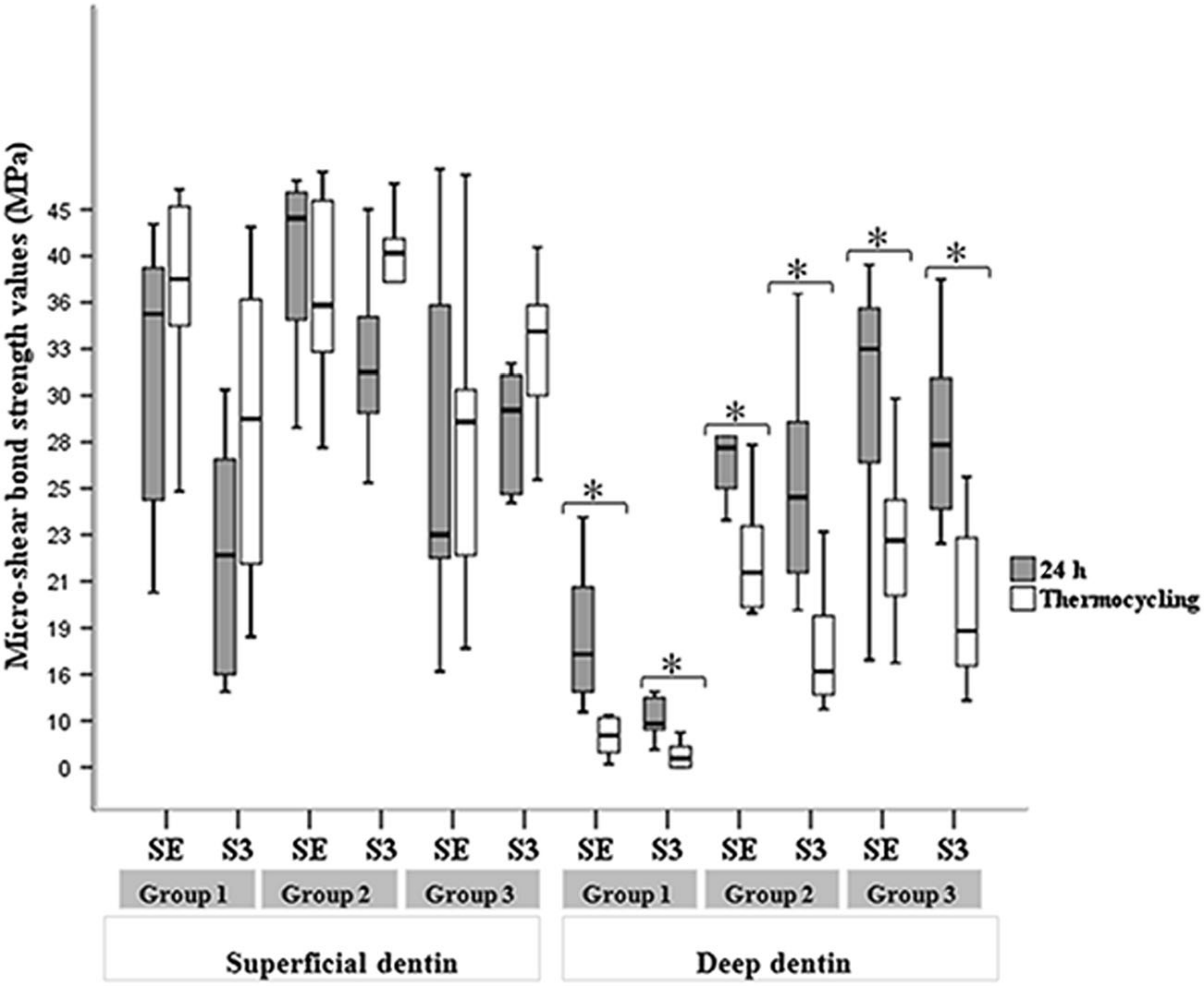
Box plots of micro-shear bond strengths to superficial and deep dentin at 24 h and after thermocycling. *Asterisk* indicates statistical significance (*p* < 0.05). *SE* Clearfil SE bond, *S3* Clearfil S^3^ bond

After thermocycling, no significant difference was found among the bond strengths of the superficial-dentin subgroups (*p* = 0.12). However, the bonding strength to deep dentin significantly improved for each adhesive with laser treatments (Group II and III) when compared to the control group (Group I) (*p* < 0.004). For each adhesive agent used, the bond strength to deep dentin was significantly lower than that to superficial dentin regardless of surface treatment after thermocycling (*p* < 0.02). The bond strength to superficial dentin was stable after thermocycling for all subgroups, whereas the bond strength to deep dentin decreased significantly after thermocycling for all subgroups (Fig. 2).

The chi-square test showed no significant differences in the failure mode after the aging procedure for any of the subgroups and within the subgroups of deep or superficial dentin (*p* > 0.05). Under thermocycling, four spontaneous debondings on the deep-dentin surface were observed for Clearfil S^3^ Bond in Group I; these failures were considered as 0 MPa.

## Discussion

In the present study, we examined whether laser surface conditioning in superficial and deep dentin would either improve or harm the bond stability of self-etch adhesive bonding agents as compared to dentin surface flattened with sandpaper. Bonding stability between the restorative material and tooth substrate is an important factor in the quality of the filling and the subsequent clinical success of restoration. Several methods can be used to evaluate bonding stability. In the present study, thermocycling for 15,000 cycles was chosen as the in vitro aging method (over, for example, a long period of immersion in water) due to the advantageous shorter testing time.

According to the findings of this study, the bond strengths in superficial dentin groups increased or were stable from the period of time 24 h after bonding through 15,000 thermal cycles. The heat change in the thermal cycle may accelerate post-cure polymerization of the adhesive by increasing the conversion rate of monomers, which causes improvement in bond strengths. Par et al. reported that a temperature increase was correlated with a significant increase in the degree of post-cure polymerization [21]. In addition, the adhesives used in this study include methacryloxydecyl phosphate (MDP). This monomer contains phosphate groups that provide chemical bonds with hydroxyapatite in tooth substrate. It has been asserted that the quality of chemical bonding with MDP monomer might be time-dependent. Bonding agents containing MDP have been proven to form nanolayering structures that appear very stable according to their low dissolution rate in water [22].

In the present study, the bond strength to laser-irradiated superficial dentin did not statistically differ from the bond strength with sandpaper-abraded superficial dentin after thermocycling, regardless of the adhesive agent used. A few studies exist that reviewed laser-irradiated dentin adhesive bond stability. Akin et al. examined the performances of all-in-one bonding agents applied to an occlusal cavity prepared using an Er:YAG laser (200 mJ, 10 Hz) with output power of 2 W after water storage for 6 months and thermocycling (10,000 cycles). In this study, the performance of the respective bonding agents was not affected by the aging methods [20], consistent with our results in superficial dentin. Contrary to this result, Amaral et al. noted that the bond strength of an etch-and-rinse adhesive system to the cavity prepared with Er:YAG laser was negatively influenced by aging procedures [19].

The results of the current study demonstrate that the bond strengths in superficial dentin groups were significantly higher than in deep-dentin groups after thermocycling. Furthermore, the bond strengths in the deep-dentin groups significantly decreased after the thermocycling process, indicating the degradation of resin–dentin bonds. The degraded and decreased bonding in deep dentin may relate to its special structure. The higher diameter and density of tubules in deeper dentin result in more wetness with the preparation of deep dentin compared to that of superficial dentin. The larger tubule orifices in deep dentin may cause severe contamination by water where the adhesive and dentin are bonded. Excessive water at this interface may interfere with polymerization and the infiltration of adhesive monomers and also cause phase separation of the bonding agent, resulting in nanoleakage and impaired mechanical properties [23, 24]. Thermocycling could also activate the dentinal matrix metalloproteinases, of which there was a high level in the deep dentin and cause the degradation of collagens in the hybrid layer [25]. Previous studies have reported lower bond strengths to deep dentin than to superficial dentin.

In the present study, higher bond strength existed for the adhesives used in laser-irradiated deep dentin when compared to the bond strength of the control group after thermocycling. Based on these findings, the null hypothesis tested should be partially rejected. With laser treatment, intertubular dentin is selectively ablated more than the peritubular dentin, creating a cuff-like appearance with protrusion of the dentinal tubules. Laser treatment also has the potential to increase the bonded surface area. The absence of a smear layer and the opening of the dentinal tubules are additional factors that may improve adhesion to laser-irradiated dentin [17].

Higher bond strength to laser-irradiated deep dentin might be explained by the mechanical adhesion provided by resin tag formation and the infiltration of the bonding agent into the created porosities. Laser irradiation can provide better infiltration into intratubular dentin with resin tags of adhesives. Polymerized resin tags form a plug to seal the irradiated dentin tubules and in particular can prevent any movement of fluids in the deep dentin [26]. One study has reported that resin tags were more pronounced in dentin prepared using a laser than in that prepared using a bur, regardless of the adhesives applied [27]. Visuri et al. determined that the bonding strengths to dentin irradiated with an Er-YAG laser were higher than those obtained with acid etching [17]. On the other hand, another study has reported that the remaining denatured collagen fibrils in the laser-treated dentin layer were fused and poorly connected to the underlying dentin tissue, and that the presence of this fused layer can limit diffusion of resin monomers into the subsurface intertubular dentin, causing a decrease in resin–dentin bond strength [28]. However, Benazzato and Stefani stated that normal collagen fibers in the more superficial region of dentinal tubules were found, and that adhesive materials may create a hybrid layer in the superficial region [29]. In this study, laser treatment did not affect the bonding strength to superficial dentin when compared to the control group. The increase of bonding strength to deep dentin with laser treatment may be explained by several factors, including tooth age, structural variations in dentin depth, changes in dentin permeability, and composition of the smear layer. There is no information concerning the ultimate effect of laser irradiation on different dentin depths, and further study is required to evaluate the adhesive–dentin interface at different depths of laser-irradiated dentin.

Several studies have reviewed the influence of pulse frequency and output energy on bond strength. However, the precise laser settings and parameters that provide optimal resin–dentin bond strengths have not been determined. Gonçalves et al. reported that the increased pulse frequency of the Er:YAG laser (with 80 mJ; 1, 2, 3, and 4 Hz) significantly decreased resin–dentin bond strength [30]. Aizawa et al. found significant differences among groups treated with 100 mJ-10, 50 mJ-20, and 33 mJ-30 Hz and observed that dentin surfaces treated with 100 mJ-10 Hz provided significantly higher bond strength than that of the other groups [31]. Monghini et al. stated that no significant differences were found among the bond strengths of dentin specimens treated with 60, 80, and 100 mJ at 2 Hz [32].

The differences in bond strength to irradiated dentin have usually been attributed to the different chemical or physical natures of the denatured dentin layer. In a previous study, it has been suggested that the size of the denatured dentin layer could depend on the output energy of the Er:YAG laser, while the quality could depend on the pulse frequency of the laser [31]. Increases in the pulse repetition rate lower the pulse energy of the emitted laser pulse, which might provide a reduction in mechanical damage but also result in increased thermal effects [33]. Sheth et al. reported that shorter laser pulses provided better bonding to dentin of composite materials because thermal damage was minimized [34]. Consequently, the effects of various parameters of the Er:YAG laser on dentin structure, particularly on the collagen fiber network, have not been clarified.

In this study, we chose a laser setting of 1.2 W, as recommended for dentin surface conditioning by the manufacturer, and a lower output level of 0.5 W, in order to prevent excessive tissue damage. However, the results of the present study showed that there was no significant difference between these different laser settings. The exposure of the dentin surface to the higher laser energy might easily cause more profound morphological changes, particularly to dental pulp damage in deep dentin, because of the heat accumulation and tissue ablation compared to lower laser energy. Therefore, the minimum energy that could achieve the optimal resin–dentin bond strength must be used for dentin surface etching.

In the present study, we evaluated the bond strengths of two different self-etch bonding agents to dentin prepared by sandpaper or Er:YAG laser. After thermocycling, Clearfil SE Bond had bonding strength to deep dentin higher than that of Clearfil S3 Bond in all groups, but this difference was not statistically significant. The acidic monomer found in self-etch adhesives demineralizes the superficial dentin surface by partially dissolving minerals around the collagen fibrils and simultaneously allowing infiltration of resin monomers [35]. The acidic functional monomers produced a potential chemical bond with hydroxyapatite crystals [36]. Another important advantage of a mild self-etch adhesive is the preservation of some hydroxyapatite crystal around collagens, protecting the collagen fibrils against hydrolysis [37]. To date, one-step self-etch adhesive agents have usually shown lower bond strength than have multi-step adhesive agents. The inferior bonding effectiveness of one-step self-etch adhesive agents depends on factors including the conversion rate of resin monomers, reduced mechanical properties of the resulting polymer, and phase separation [38].

Some researchers have believed that dentin-bonding materials do not routinely seal the pulpal floor [39]. Self-etch adhesives have been shown to have significantly lower bond strength to deep dentin with a tubule orientation perpendicular to the surface [5]. The spontaneous debondings during thermocycling in this study were observed for Clearfil S^3^ Bond applied to deep-dentin specimens of the control group. de Souza et al. reported that the bond strength to deep dentin increased when non-rinsing conditioner was used after laser treatment [40]. This increase has been attributed to the adherence of calcium ions to the hard tissue of teeth for the agent used. Some studies have reported that self-etch adhesive system produced better bonding strength to laser-irradiated dentin [15, 16].

## Conclusions

Within the limitations of this in vitro study, the following can be concluded:

- Er:YAG laser surface treatment (regardless of used parameters) did not affect the bonding strength to superficial dentin after thermocycling when compared to the control group. However, the bond strength to deep dentin after laser irradiation significantly increased when compared to the control group.
- The bond strengths measured for all superficial dentin subgroups after thermocycling were stable. The bonding strength to deep dentin decreased for all subgroups after thermocycling.
- No significant difference was found between adhesive materials used regardless of the applied surface treatment after thermocycling.
- After thermocycling, there was no significant difference between the laser parameters used.
- Significantly lower bond strength was measured after thermocycling in the groups using teeth prepared to reveal the deep dentin compared to the groups using the superficial dentin preparations.
